# Robotic-assisted partial nephrectomy (RAPN) and standardization of outcome reporting: a prospective, observational study on reaching the “Trifecta and Pentafecta”

**DOI:** 10.1007/s11701-020-01141-z

**Published:** 2020-09-03

**Authors:** D Sri, R. Thakkar, H R H Patel, J. Lazarus, F. Berger, R. McArthur, H. Lavigueur-Blouin, M. Afshar, C. Fraser-Taylor, P. Le Roux, J. Liban, C. J. Anderson

**Affiliations:** St George’s Hospital NHS Trust, Tooting, London, SW17 0QT UK

**Keywords:** Robot-assisted partial nephrectomy, Partial nephrectomy, Trifecta, Pentafecta

## Abstract

Partial nephrectomy (PN) for small renal masses is common, but outcomes are not reported in a standard manner. Traditionally, parameters such as 90-day mortality, blood loss, transfusion rates, length of stay, nephrometry scoring and complications are published but their collective impact on warm ischemia time (WIT) and post-surgery GFR is rarely determined. Thus, our aim was to assess if “Trifecta” and “Pentafecta” outcomes could be used as useful surgical outcome markers. A prospective database of 252 Robotic-Assisted PN (RAPN) cases (2008–2019) was analysed. “Pentafecta” was defined as achievement of “Trifecta” (negative surgical margin, no postoperative complications and WIT of < 25 min) plus over 90% estimated GFR preservation and no CKD stage upgrading at 1 year. Binary logistic regression analysis was conducted to predict factors which may prevent achieving a Trifecta/Pentafecta. Median tumour size was 3 cm and mean WIT was 15 min. Positive surgical margins (PSM) occurred in 2 cases. Overall, the intra-operative complication rate was 7%. One recurrence conferred 5-year cancer-free survival of 97%. Trifecta outcome was achieved in 169 (67%) and Pentafecta in 141 (56%) of cases. At logistic regression analysis, intraoperative blood loss was the only factor to affect Trifecta achievement (*p* = 0.018). Advanced patient age negatively impacted Pentafecta achievement (*p* = 0.010). The Trifecta and Pentafecta outcomes are easily applicable to PN data, and offer an internationally comparable PN outcome, quality measure. We recommend applying this standardization to national data collection to improve the quality of reporting and ease of interpretation of surgeon/centres’ outcomes.

## Introduction

The modern approach to small renal masses (SRM) has evolved over the last two decades, with mandatory discussions required by a decision-making multidisciplinary team (MDT), including urological surgeons, radiologists, pathologists and oncologists [1]. Progress in medical technology has enabled an improvement in diagnostics and treatments available for SRMs, which has seen a shift in the standard of care to favour nephron-sparing surgery (NSS) [2]. This is reflected in international guidance, which recommends NSS in the treatment of renal masses < 4 cm [2, 3].

Treatment options widen when SRMs are amenable to minimally invasive percutaneous ablative therapies (e.g. Percutaneous Cryotherapy or Radiofrequency ablation) [4] or NSS (e.g. RAPN/Laparoscopic partial nephrectomy/Laparoscopic-assisted ablative therapies) [5–7]. In most centres, a renal mass would be characterized by a dedicated renal-protocol CT scan/MRI and staged with a Chest CT. This would then be discussed at an MDT where the PADUA/RENAL nephrometry score and patient performance status can be considered to provide the optimal treatment decision for the patient.

Oncological outcomes for NSS (i.e. partial nephrectomy) have been shown to be comparable to radical nephrectomy [8]. Laparoscopic partial nephrectomy (LPN) is widely considered a difficult procedure, with a long learning curve to master. This contrasts with RAPN, whose advocates cite enhanced visibility, greater manoeuvrability, quicker and easier suturing techniques, reduced tremor amongst others to provide a shorter learning curve [9].

Recent interest in standardizing outcomes of RAPN has led to the terms ‘Trifecta and Pentafecta’ achievements. Trifecta describes achieving no complications, negative surgical margin, and warm ischemia time (WIT) < 25 min [10] and Pentafecta adds information on renal function preservation (90% of pre-operative eGFR) and avoidance of upstaging of a patient’s chronic kidney disease status [11].

Traditionally, parameters such as 90-day mortality, blood loss, transfusion rates, length of stay, nephrometry scoring and complications are published but their collective impact on functional outcome is rarely determined. Thus, we aimed to assess if “Trifecta” and “Pentafecta” outcomes could be used as useful surgical outcome markers.

## Patients and methods

From a prospectively collected database of 1700 renal cases, we collected 252 patients with SRMs who underwent RAPN by a single surgeon at our institution. This cohort was derived from the inception of our robotic renal program in 2008 to the present time. RAPN was performed via a trans-peritoneal approach using the da Vinci Surgical System (Intuitive Surgical, Sunnyvale, CA, USA). Patients underwent intraoperative ultrasound-guided excision with two-layer renorrhaphy. Although during our early experience with RAPN main arterial clamping was utilised as standard, it has now become our default practice to perform selective arterial clamping. Patients were discussed at MDT and post-operatively were risk stratified based on their Liebovich score and followed up accordingly. All patients were followed up in our tertiary centre based on their risk stratification, via a combination of outpatient visits and telephone clinics.

Patient demographics including age, BMI, preoperative renal function, ASA grade/Charlson co-morbidity score and chronic kidney disease stage were recorded and analysed. Renal tumour characteristics were summarised using the Preoperative Aspects and Dimensions Used for an Anatomical (PADUA) score [12]. Intraoperative data including operative time, training time, warm ischemia time (WIT), estimated blood loss (EBL) and post-operative pathology were assessed. Renal function was assessed based on eGFR.

Local and distant recurrence rates, and cancer-free survival were our primary oncological outcome measures. We utilised trifecta achievement (negative surgical margin, no postoperative complications and a WIT of ≤ 25 min) as an early functional outcome measure and pentafecta achievement (trifecta, 90% of pre-op eGFR and no CKD stage upgrading at 12 months post-op) as a long-term functional outcome measure. Multivariate analysis was carried out to determine predictors that adversely impact trifecta and pentafecta achievement in our cohort. Data analysis and survival curve construction were performed using SPSS v25.

## Results

### Demographics and lesion characteristics

To date a total of 260 patients have undergone RAPN, however, in the interest of at least 1 month follow-up only the first 252 patients have been included in this study (Table 1). Our cohort’s mean age ± SD of 58 ± 12 years and were predominantly male (ratio of 1.8:1). They had a mean BMI ± SD of 29 ± 4.7 and a median American Society of Anaesthesiologists (ASA) score of 2. Twenty-seven (11%) of cases had pre-existing CKD stage 3/>, and 31 patients underwent RAPN for imperative indications.

**Table 1:** Patient demographics

	No.	%/(range)
Total no. of cases	252	
Median age	58	(17–86)
Female	89	35%
Male	163	65%
Laterality		
Right	138	55%
Left	114	45%
Median BMI	28.2	(19.2–41)
Median ASA	2	(1–3)
Median eGFR	60	(28–60)
CKD stage 3/>	27	11%
Imperative indication (solitary kidney, bilateral disease, CKD)	31	12%

Our median lesion size was 3 cm on pre-operative imaging (Table 2), with a mean PADUA score of 8. Following PADUA risk stratification, 60% of patients had intermediate or highly complex lesions (PADUA scores 8–12). Pre-operative biopsy was utilized in 18% of the cases.

**Table 2:** Lesion characteristics

	No.	%/(range)
Median tumour size	2.9 cm	(1 cm–7 cm)
Median Padua Score	8	(6–12)
Low PADUA risk (6–7)	87	39%
Intermediate PADUA risk (8–9)	91	41%
High PADUA risk (10–12)	46	21%
Renal Sinus Involvement	69	30%
Exophytic > 50%	107	47.8
Exophytic < 50%	85	37.9
Endophytic	32	14.3
Anterior lesions	125	56%
Posterior lesions	99	44%
Pre-op biopsy	33	15%

### Operative characteristics and complications

We favoured the trans-peritoneal approach to RAPN, with a mean operating time ± SD of 165 ± 48 min (Table 3). Our mean WIT ± SD was 15 ± 6 min, and our median blood loss was 250 mls. We utilized super-selective clamping in 50% of cases. There was one conversion to open surgery for cyst rupture and another converted to hand-assisted laparoscopic approach due to dense adherent perinephric fat. One patient underwent conversion to radical nephrectomy in our series, secondary to anaesthetic complications resulting in an on-table cardiac arrest.

**Table 3:** Intraoperative characteristics

	No.	%/(range)
Retroperitoneal	5	2.2
Transperitoneal	219	97.8
Total conversion	2	
To open secondary to tumour rupture	1	
To hand-assisted lap secondary to dense fat	1	
Median op time	165 min	(60 min–293 min)
Mean training time	14 min	(10 min–180 min)
ICG used	132	59%
Median blood loss	200 mls	(0 mls–2500 mls)
Super-selective clamping	104	46%
Median Warm Ischaemic Time	15 min	(5 min–35 min)

Our overall intraoperative complication rate was 7% (Table 4). Our mean ± SD length of stay was 4 ± 2.9 days and our overall postoperative complication rate was 31%; 19% of these were Clavien 3 or higher in severity.

**Table 4:** Complications

	No.	%/(range)
Intraop complications	16	7%
Vascular injury	10	
Anaesthetic	2	
Leak	1	
Port site bleeding	2	
Tumour rupture	1	
Post op complications	74	33%
Clavein 3/>	15	20%
Bleeding	14	19%
Infection (chest, woung, urine)	19	26%
Ileus	12	16%
Urine leak	4	5%
Median length of stay	4 days	(1–35 days)

### Pathology and outcome

Seventy-eight percent of lesions were malignant (Table 5), with clear cell RCC the predominant histological subtype (68%). Of the 22% benign histology in our cohort, oncocytoma were the commonest subtype (55%), followed by AMLs (27%). Sixteen cases were stage T3a due to microscopic fat invasion, with pT1a tumours accounting for the majority of cases (82%). We report a positive surgical margin in just 2 patients with an overall rate of less than 1%.

**Table 5:** Pathology outcome

	No.	%/(range)
Histology		
Benign	50	22%
Oncocytoma	27	54%
AML	13	26%
Malignant	174	78%
Clear Cell RCC	119	68%
Papillary RCC	31	18%
Grade		
Fuhrman G1-G2	118	68%
Fuhrman G3-G4	43	32%
T Stage		
pT1a	144	83%
pT1b	17	10%
pT3a	10	7%
Positive surgical margin	2	1%

**Fig. 1:**
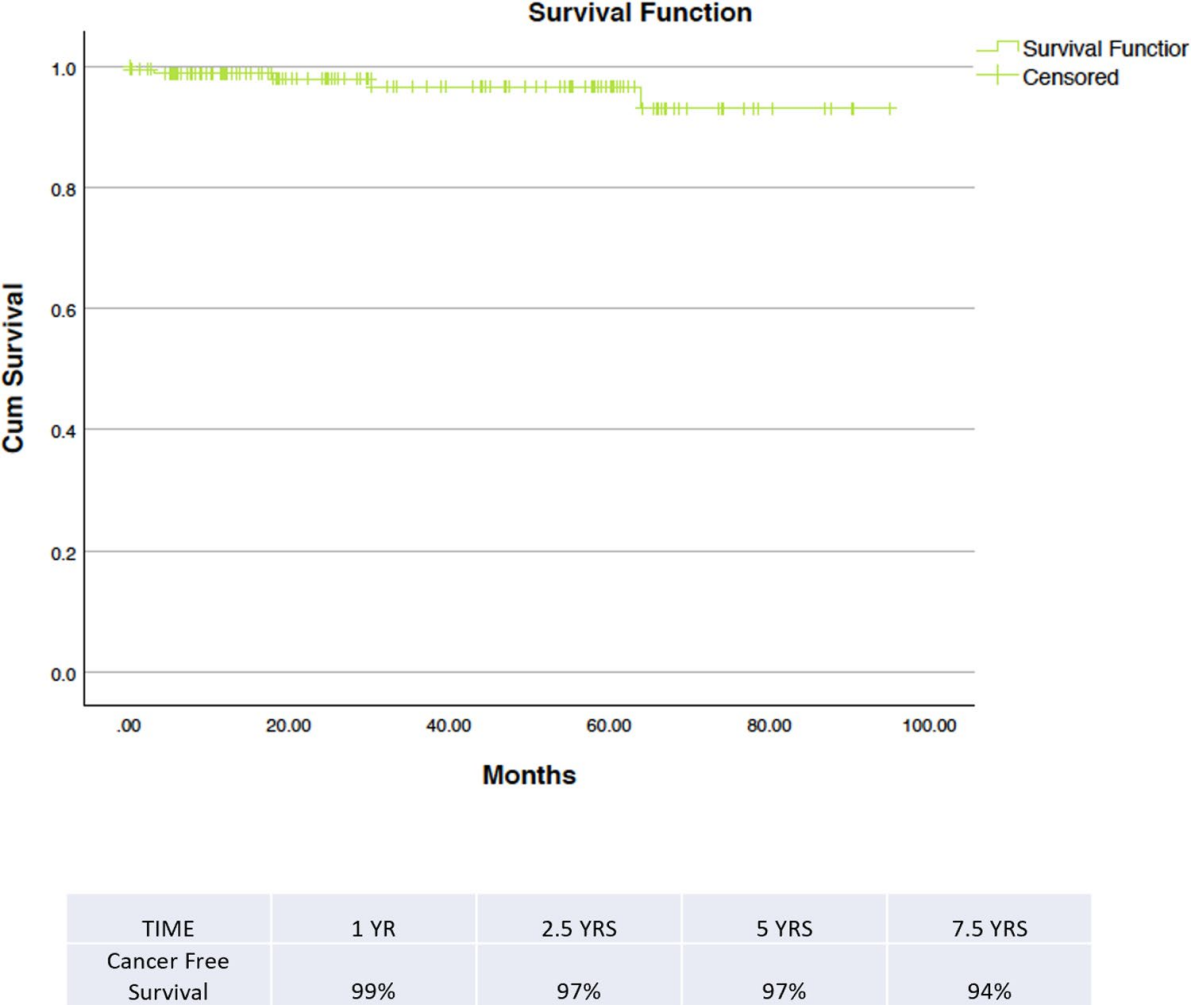
Kaplan–Meier curve demonstrating cancer-free survival in our cohort

The median follow-up amongst our malignant cohort of patients was 18 months (range 0–95 months). We had a single case of recurrence in that time (Table 6), giving an overall recurrence rate of 0.3%. There were no deaths attributable to renal malignancy in our series (outcomes determined by death certification). Our cancer-free survival at 1 year was 99% and at 5 years was 97% (Fig. 1).

**Table 6:** Oncological/functional outcome

	No.	%/(range)
Median follow-up	25 Months	(2–95 months)
Recurrence	1	0.50%
Deaths		
All causes	4	2%
Due to RCC	0	
Trifecta achievement	145	64%
Pentafecta achievement	122	54%

Twenty-seven patients (11%) had an upstage in their CKD status at 12 months post-op. We achieved a trifecta outcome in 67% of cases and a pentafecta outcome in 56% of cases. Multivariate analysis of our series demonstrated no significant determinants of not being able to achieve a trifecta outcome, with age the only predictor of failing to achieve a pentafecta outcome (Table 7).

**Table 7:** Binary logistic regression analysis of factors affecting failure to achieve pentafecta

Factor	Exp(b)/odds ratio	Sig
Blood loss	0.998	0.061
ASA	0.0593	0.142
T1a	0.477	0.110
T1b	0.440	0.252
HTN	0.946	0.890
Diabetes	1.943	0.210
Age	1.021	0.010*
Tumour size	0.872	0.452
WIT	1.033	0.415

Asterisk (*) denotes statistical significance

## Discussion

We present our decade long experience with RAPN at a tertiary referral centre in the UK, in achieving both good oncological and functional outcomes for our patients. Our PSM rate was < 1%, with a solitary recurrence and 5-year cancer-free survival rates of 97%. Our PSM rates are lower than those in the published literature for RAPN. Although there can be oncological uncertainty in the context of a PSM, there is debate regarding its significance. In a retrospective multi-institutional analysis of 1240 patients, 97 of whom had a PSM, Shah et al. demonstrated low-risk T1a tumours had a similar recurrence-free survival rate to having negative surgical margins. High-risk, on the other hand, had a 45% risk of recurrence in 5 years [13]. No recurrences were identified amongst 31 PSM patients in a retrospective study of 1831 pT1 RCCs after 32.5 months follow-up [14]. Both our PSM patients fit the low-risk category and they remain recurrence free at 59 months and 62 months follow-up, respectively.

Studies have shown that a third of SRMs < 2.5 cm in size are benign, whilst 8% of lesions > 4 cm are benign. This increases further in younger patients, where 44% of SRMs < 2.5 cm are benign [15]. We do not routinely utilise biopsy pre-operatively, with it reserved for imperative indications (solitary kidney, bilateral disease, CKD); usually where there is another cancer present in other organs and where there is diagnostic uncertainty on imaging. Our overall benign histology rate is 22% across our series and 20% for masses < 4 cm (*n* = 42). This rises to 28% for masses < 2.5 cm (*n* = 26). We have, therefore, seen a change in our practice, particularly utilising pre-operative biopsy on an increasing basis in SRMs < 3 cm.

Our major complication rate (Clavein 3/>) of 19% is in line with other large series and studies on RAPN, whilst our open conversion rates (< 1%) are lower than those reported elsewhere [5, 9]. Only 1 patient (0.04%) was converted to open procedure (due to cystic tumour rupture), whilst another patient had his procedure completed using hand-assisted laparoscopic techniques. In our experience, other intra-operative complications such as vascular injury and urine leaks were successfully dealt with using robotic techniques.

Although the primary goal of NSS is oncological control, the secondary goal is maximising post-operative renal function. We utilise selective arterial clamping (SAC) in our practice where feasible (50%), coupled with ICG administration to ensure adequate ischaemia has been achieved. The rationale behind this approach is that the limitation of global ischaemia to the kidney reduces the ischaemic damage and improves the long-term functional outlook. Our median WIT was 15 min, with only 9 patients having a WIT of > 25 min (3.5%). There remains some controversy in the literature regarding the effectiveness of SAC. Retrospective studies have shown statistically significant preservation of eGFR in immediate post-operative period following SAC; however, this becomes insignificant at 3- and 6-months post operatively [16, 17]. These studies are further limited by a small sample size (42 and 25 selectively clamped cases, respectively), variability in WIT and pre-operative renal function across the two groups. Paulucci et al. conducted a multi-institution prospective study comparing main arterial clamping (MAC) to SAC in patients that were matched for age, sex, BMI, ASA, RENAL nephrometry score, tumour size, WIT and baseline eGFR. They found that in 132 patients undergoing MAC relative to 66 SAC patients, no statistically significant difference in the incidence of AKI in the first 30 days (*p* = 0.315) and no difference in reduction in eGFR (*p* = 0.518) or progression to CKD Stage 3/> (*p* = 0.792) at median 7.5 months follow-up [18]. In our cohort, unmatched analysis suggests no statistically significant difference in  % change in eGFR at 12 months post-procedure between the MAC and SAC group (eGFR drop of 3% vs 2%, *p* = 0.618).

The trifecta outcome measure introduced by Hung et al., has been adopted in some studies as a surgical outcome measure. It comprises negative surgical margins, no post-operative complications and a WIT of < 25 min [10]. The more recent pentafecta outcome measure which in addition to the trifecta achievement requires renal function preservation (90% of pre-operative eGFR) and avoidance of upstaging of a patient’s chronic kidney disease status, allows for a better understanding into the achievement of the secondary goal of maximising post-operative renal function [11]. Our trifecta and pentafecta rates were 67% and 56%, respectively. No patients required dialysis post procedure, and over 90% of our patients remained in the same CKD stage category post-operatively. Studies that look at RAPN focus on and explore the individual parameters that make up the trifecta and pentafecta, in the context of their overall outcomes and learning curves. Until recently, however, studies seldom report the rate of trifecta and pentafecta achievement. Similarly, the national reporting of partial nephrectomy outcomes in the UK follows suit with their collective impact on achieving the trifecta/pentafecta for a patient not recorded. Where reported, there is variation in the achievement of trifecta outcomes (32%–81%); this is somewhat confounded by a lack of standardisation in the definition of trifecta achievement, with some studies utilising a WIT of < 20 min and Clavein–Dindo ≥ 2 complications when assessing trifecta achievement [19]. Allowing for these variations our trifecta rates hold us in good stead when compared with other similar studies.

Reporting of pentafecta outcomes is also a contemporary practice with only a few studies that deal with this particular functional outcome measure in the context of RAPN. Table 8 summarises pentafecta outcomes amongst current studies.

**Table 8:** Comparison of studies on pentafecta outcomes in RAPN

Series	Year	No. of Patients	Tumour Size	Pentafecta
Kim et al. [19]	2016	120	T1a (2.6 cm) T1b (5 cm)	T1a = 38% T1b 27%
Stroup et al. [24]	2017	404	Retroperitoneal (2.9 cm) Transperitoneal (3.1 cm)	Retroperitoneal = 43% Transperitoneal = 34%
Kang et al. [25]	2017	362	2.9 cm	34%
Castelluci et al. [26]	2019	123	< 4 cm/> 4 cm cohorts	< 4 cm = 23%; > 4 cm = 11%
Current study	2019	224	2.9 cm	54%

Generally, our reported rates of pentafecta achievement are superior to those in the literature, although these aren’t propensity-matched patients to allow for a fair comparison. From a technical standpoint along with aiming to adhere to surgical principles of shorter surgical time, low WIT, minimise blood loss, we work closely with our anaesthetic team to ensure good intra and post-operative hydration of our RAPN patients, supplemented with pre-clamping Mannitol instillation to ensure a well-perfused kidney prior to subjecting it to any ischaemic insult. This may be contributory to the good functional outcomes that we have demonstrated in our study. The use of the osmotic diuretic Mannitol in NSS is controversial with its use initially based on clinical experience and animal studies. The Urology service at the Memorial Sloan Kettering Cancer Centre retrospectively looked at 285 patients undergoing PN and found the similar recovery of renal function at 6 months in patients that received intravenous Mannitol vs those that did not [20]. In 2018 the same group published the results of a prospective double-blind trial where 199 patients with normal renal function undergoing PN were randomised to receiving either mannitol or a placebo. They found no statistically significant difference between the renal function of both groups at 6 months, although the study only looked at patient’s with normal renal function [21]. A retrospective analysis by Omae et al. explored the effect of peri-operative mannitol in open PN of solitary kidneys. They found no statistically significant difference in the mean eGFR at 1 day, 1 month, 3 months and 6 months post operatively in the 20 patients who received mannitol compared to the 35 patients that did not [22]. There seems to be a shift in consensus towards discontinuing the use of Mannitol in patients with normal pre-operative renal function.

In our series logistic regression analysis suggested intraoperative blood loss was the only factor to significantly affect Trifecta achievement (*p* = 0.018). Advanced patient age was found to be statistically significant (*p* = 0.010) in determining failure to achieve pentafecta outcomes. There is again variability in trying to determine factors that may adversely influence the achievement of these outcomes in the literature. A multicentre study assessing 1222 RAPN found tumour size (*p* < 0.001) and hospital case volume (*p* = 0.005) to be the factors that most influenced trifecta achievement [23], whilst an assessment of 277 RAPN cases found R.E.N.A.L nephrometry scoring to be a significant predictor of achieving pentafecta outcome [19]. In our series, both tumour size (*p* = 0.980) and nephrometry scores (*p* = 0.880) were considered to be statistically insignificant, which is supported by other series [24–26]. Stroup et al. identified no statistically significant difference in surgical approach either with pentafecta outcome (*p* = 0.526), although the approach was based on technical considerations, rather than in a randomised fashion.

Our study is not without its limitations. Although our database is prospectively recorded, the analysis carried out is retrospective. The single surgeon, single centre nature of this study can limit its application to other centres. Based on our study design and database recording it is also difficult to adjust for the experience of assistants and training time during the analysis of various outcome measures. The analysis represents the entire RAPN experience of a single surgeon who was an early adopter of the technique at the commencement of the series in 2008. Hence the series represents initially an evolution of RAPN technique and subsequently a well-established high-volume operation. At inception, full renal arterial clamping was performed and the renorraphy evolved over time with changes in the suture material and technique. After being the first surgeon in the UK to use ICG for selective arterial clamping, this subsequently became the preferred approach in RAPN at our centre.

## Conclusion

Studies demonstrating oncological safety and preserving functional outcome have been integral in the evolution of NSS. We demonstrate comparable outcomes post RAPN with conventionally reported parameters (e.g. PSM, recurrence, WIT). We report similar overall trifecta (67%) achievement to other series and better than previously reported pentafecta (56%) rates in our series. We feel that reporting pentafecta achievement would better facilitate the assessment of long-term functional outcome post RAPN. These outcomes are easily applicable to UK PN data and offer an internationally comparable PN outcome measure. We recommend applying this standardization to national data collection to improve the quality of reporting surgical outcomes.

## Data Availability

Available on request.

## References

[1] https://www.baus.org.uk/_userfiles/pages/files/Publications/MDTRenalCancerGuidance.pdf. Accessed on June 2019

[2] http://uroweb.org/guideline/renal-cell-carcinoma/#7. Accessed on June 2019

[3] www.auanet.org/guidelines/renal-mass-and-localized-renal-cancer-new-(2017). Accessed on June 2019

[4] Finelli A, Ismaila N, Bro B (2017). Management of small renal masses: American Society of Clinical Oncology Clinical Practice Guideline. J Clin Oncol.

[5] Aron M, Koenig P, Kaouk JH, Nguyen MM, Desai MM, Gill IS (2008). Robotic and laparoscopic partial nephrectomy: a matched pair comparison from a high-volume centre. BJU Int.

[6] Gill IS, Kavoussi LR, Lane BR, Blute ML, Babineau D, Colombo JR, Frank I, Permpongkosol S, Weight CJ, Kaouk JH, Kattan MW, Novick AC (2007). Comparison of 1,800 laparoscopic and open partial nephrectomies for single renal tumors. J Urol.

[7] Kunkle D, Uzzo R (2008). Cryoablation or radiofrequency ablation of the small renal mass: a meta-analysis. Cancer.

[8] Kim SP, Thompson RH, Boorjian SA (2012). Comparative effectiveness for survival and renal function of partial and radical nephrectomy for localised renal tumours: a systematic review and meta-analysis. J Urol.

[9] Choi JE, You JH, Kim DK, Rha KH, Lee SH (2015). Comparison of perioperative outcomes between robotic and laparoscopic partial nephrectomy: a systematic review and meta-analysis. Eur Urol.

[10] Hung AJ, Cai J, Simmons MN, Gill IS (2013). “Trifecta” in partial nephrectomy. J Urol.

[11] Zargar H, Allaf ME, Bhayani S, Stifelman M, Rogers C, Ball MW (2015). Trifecta and optimal perioperative outcomes of robotic and laparoscopic partial nephrectomy in surgical treatment of small renal masses: a multi-institutional study. BJU Int.

[12] Ficarra V, Novaro G, Secco S (2009). Preoperative aspects and dimensions used for an anatomical (PADUA) classification of renal tumours in patients who are candidates for nephron-sparing surgery. Eur Urol.

[13] Shah PH, Moreira DM, Okhunov Z (2016). Positive surgical margins increase risk of recurrence after partial nephrectomy for high risk renal tumors. J Urol.

[14] Kang HW, Lee SK, Kim WT, Yun SJ, Lee SC, Kim WJ, Hwang EC, Kang SH, Hong SH, Chung J, Kwon TG, Kim HH, Kwak C, Byun SS, Kim YJ, KORCC (KOrean Renal Cell Carcinoma) Group (2016). Surgical margin does not influence recurrence rate in pT1 clear cell renal cell carcinoma after partial nephrectomy: a multicenter study. J Surg Oncol..

[15] Fernando A, Fowler S, O’Brien T (2016). Nephron-sparing surgery across a nation—outcomes from the British Association of Urological Surgeons 2012 national partial nephrectomy audit. BJU Int.

[16] McClintock TR, Bjurlin MA, Wysock JS (2014). Can selective arterial clamping with fluorescence imaging preserve kidney function during robotic partial nephrectomy?. Urology.

[17] Komninos C, Shin TY, Tuliao P (2015). Renal function is the same 6 months after robot-assisted partial nephrectomy regardless of clamp technique: analysis of outcomes for off-clamp, selective arterial clamp and main artery clamp techniques, with a minimum follow-up of 1 year. BJU Int.

[18] Paulucci DJ, Rosen DC, Sfakianos JP, Whalen MJ, Abaza R, Eun DD, Krane LS, Hemal AK, Badani KK (2017). Selective arterial clamping does not improve outcomes in robot-assisted partial nephrectomy: a propensity-score analysis of patients without impaired renal function. BJU Int.

[19] Kim DK, Kim LH, Raheem AA, Shin TY, Alabdulaali I, Yoon YE, Han WK, Rha KH (2016). Comparison of trifecta and pentafecta outcomes between T1a and T1b renal masses following robot-assisted partial nephrectomy (RAPN) with minimum one year follow up: can RAPN for T1b renal masses be feasible?. PLoS One..

[20] Power NE, Maschino AC, Savage C, Silberstein JL, Thorner D, Tarin T, Wong A, Touijer KA, Russo P, Coleman JA (2012). Intraoperative mannitol use does not improve long-term renal function outcomes after minimally invasive partial nephrectomy. Urology.

[21] Spaliviero M, Power NE, Murray KS, Sjoberg DD, Benfante NE, Bernstein ML, Wren J, Russo P, Coleman JA (2018). Intravenous mannitol versus placebo during partial nephrectomy in patients with normal kidney function: a double-blind, clinically-integrated, randomized trial. Eur Urol.

[22] Omae K, Kondo T, Takagi T, Iizuka J, Kobayashi H, Hashimoto Y, Tanabe K (2014). Mannitol has no impact on renal function after open partial nephrectomy in solitary kidneys. Int J Urol.

[23] Peyronnet B, Tondut L, Bernhard JC, Vaessen C, Doumerc N, Sebe P, Pradere B, Guillonneau B, Khene ZE, Nouhaud FX, Brichart N, Seisen T, Alimi Q, Beauvalò J-B, Mathieu R, Rammal A, de la Taille A, Baumert H, Stephane D, Bruyere F, Roupret M, Mejean A, Bensalah K, The members of the French Committee of Urologic Oncology (CCAFU) (2018). Impact of hospital volume and surgeon volume on robot-assisted partial nephrectomy outcomes: a multicentre study. BJU Int.

[24] Stroup SP, Hamilton ZA, Marshall MT, Lee HJ, Berquist SW, Hassan AS, Beksac AT, Field CA, Bloch A, Wan F, McDonald ML, Patel ND, L’Esperance JO, Derweesh IH (2017). Comparison of retroperitoneal and transperitoneal robotic partial nephrectomy for Pentafecta perioperative and renal functional outcomes. World J Urol.

[25] Kang M, Gong IH, Park HJ, Sung HH, Jeon HG, Jeong BC, Jeon SS, Lee HM, Choi HY, Choi HY, Il Seo S (2017). Predictive factors for achieving superior pentafecta outcomes following robot-assisted partial nephrectomy in patients with localized renal cell carcinoma. J Endourol.

[26] Castellucci R, Primiceri G, Castellan P, Marchioni M, D’Orta C, Berardinelli F, Neri F, Cindolo L, Schips L (2018). Trifecta and pentafecta rates after robotic assisted partial nephrectomy: comparative study of patients with renal masses < 4 and ≥ 4 cm. J Laparoendosc Adv Surg Tech A.

